# Nanobody-based sandwich reporter system for living cell sensing influenza A virus infection

**DOI:** 10.1038/s41598-019-52258-7

**Published:** 2019-11-04

**Authors:** Jiali Cao, Nicole Zhong, Guosong Wang, Mingfeng Wang, Baohui Zhang, Baorong Fu, Yingbin Wang, Tianying Zhang, Yali Zhang, Kunyu Yang, Yixin Chen, Quan Yuan, Ningshao Xia

**Affiliations:** 1 State Key Laboratory of Molecular Vaccinology and Molecular Diagnostics, National Institute of Diagnostics and Vaccine Development in Infectious Diseases, School of Life Sciences, Xiamen University, Xiamen, 361102 P.R. China; 2 State Key Laboratory of Molecular Vaccinology and Molecular Diagnostics, National Institute of Diagnostics and Vaccine Development in Infectious Diseases, School of Public Health, Xiamen University, Xiamen, 361102 China; 3 Concordia International School Shanghai, 345 Huangyang Road Pudong, Shanghai, 201206 P.R. China; 4 Xiamen International Travel Healthcare Center, Xiamen, China

**Keywords:** High-throughput screening, Fluorescence imaging, Influenza virus

## Abstract

The influenza epidemic is a huge burden to public health. Current influenza vaccines provide limited protection against new variants due to frequent mutation of the virus. The continual emergence of novel variants necessitates the method rapidly monitoring influenza virus infection in experimental systems. Although several replication-competent reporter viruses carrying fluorescent proteins or small luciferase have been generated in previous studies, visualizing influenza virus infection via such strategy requires reverse genetic modification for each viral strain which is usually time-consuming and inconvenient. Here, we created a novel influenza A nucleoprotein (NP) dependent reporter gene transcription activation module using NP-specific nanobodies. Our results demonstrated the modular design allowed reporter genes (mNeonGreen fluorescent protein and Gaussia luciferase) specifically expressing to detect intracellular NP protein, and therefore acts as a universal biosensor to monitor infection of various influenza A subtypes in living cells. The new system may provide a powerful tool to analyze influenza A infections at the cellular level to facilitate new antiviral drug discovery. Moreover, this approach may easily extend to develop live-cell biosensors for other viruses.

## Introduction

According to the World Health Organization (WHO), yearly influenza epidemics are the cause of 3 to 5 million cases of severe illness, with 250,000 to 500,000 deaths each year^1^. Those who are most susceptible to contracting severe cases of influenza and/or death include pregnant women, infants, the elderly, health care workers, and those suffering from other chronic illnesses^2–7^. These highlight the limitations of current influenza vaccines and antiviral drugs. The influenza virus is a member of the Orthomyxoviridae family, and is enveloped, containing a single stranded segmented RNA genome with eight RNA segments, which are capable of encoding at least 17 proteins^8^. Two of which are hemagglutinin (HA) and nerumindase (NA), the major surface antigens of the virus^9^. Amino-acid substitutions most frequently occurred in these two proteins and therefore causes antigenic drift to generate a new influenza variant with an altered HA and/or NA that may escape from immune response stimulated by vaccination of previous virus^10–12^. Actually, because of constant variants that the virus produces, it is extremely difficult to develop a universal vaccine for influenza, and it is also hard to predict the epidemic virus strains. For this reason, the development of broadly neutralizing antibodies or other medicines which are able to counter wide ranges of the virus is necessary. Ergo, there is a need of a good cell model for robust high-throughput screening for more potent antibodies or small molecular drug candidates against influenza infection.

Fluorescence and/or bioluminescence-based viral infection reporter system enable convenient measurements of intracellular viral loads therefore provide robust tools for evaluations of antiviral agents, particularly for large-scale drug-screening. The visualization of influenza infection through fluorescence has been made possible with the development of a genetic modified influenza virus incorporating fluorescent protein expression cassette within viral genome^13–15^, which can be used in anti-Flu drug testing and antibody neutralization assay. However, the insertion of a fluorescent protein possibly affects viral infectivity. More importantly, it is difficult to make genetic modified viruses of all influenza subtypes for broad-spectrum antiviral activity test^16^. A universal method for living cell sensing influenza A virus infection of various strains will be a robust tool to facilitate both basic researches and drug developments.

For influenza virus, the nucleoprotein (NP) is a structural protein of influenza virus which encapsidates viral RNA. The NP plays an essential role in viral replication and hence becomes a promising therapeutic and diagnostic target^17^. Unlike HA and NA, NP serves as highly conserved antigenic determinants for influenza type (A, B, C). Thus, antibodies against conserved NP epitopes of influenza A virus were usually used in rapid influenza diagnosis tests (RIDTs)^18,19^. In this study, by using single domain antibodies, we developed an NP responsive reporter module for sensitive sensing influenza A virus infection. In this module, the expression of the reporter genes (green fluorescent protein and Gaussia luciferase) are dependent on the recruitment of the transactivation domain (AD) to the activating sequence upstream by DNA binding domain (DBD) via NP-specific nanobody mediated double-sandwich scaffold. We demonstrated successful living cell detections of infection and antibody neutralization of influenza A H1, H3, H5 and H7 by using this new system.

## Results

### Nanobody screening for intracellular NP-dependent EGFP transcription activiation module

**Figure 1:**
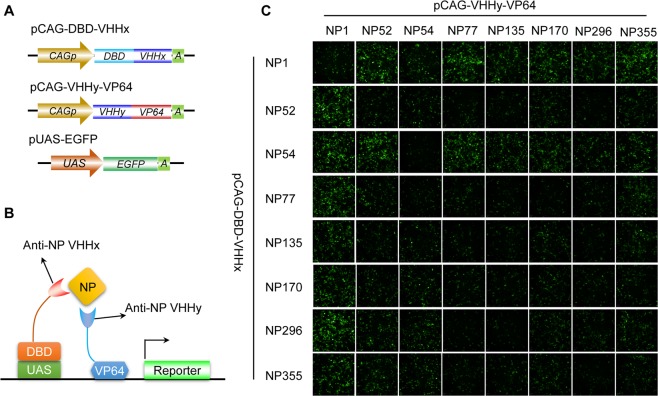
Sandwich report system based on nanobodies pair for detection of NP antigen in living cells. (**A**) The schematic diagram of cassettes for sandwich report system. (**B**) The schematic diagram showed how sandwich report system works. (**C**) Screening for nanobody pair suitable for NP detection. Different nanobody pair, report gene and NP were co-expressed in HEK293 cell. The fluorescence was detected 48 h after transfection.

In order to detect influenza A in living cells, we designed an NP-dependent reporter gene transcription activation system following the GFP-dependent transcription system as described previously^20^. The Gal4 DNA-binding domain (DBD) and VP64 activation domain (a tetrameric repeat of HSV VP16’s minimal activation domain) were separately fused to anti-NP nanobodies (VHHs) in various configurations and placed under the control of the ubiquitous CMV early enhancer/chicken β actin (CAG) promoter (Fig. 1A). In order to utilize intracellular NP protein as a dimerizer scaffolding DBD and AD domains, DBD-VHH and V16AD-VHH fusion constructs with various NP nanobodies report previously (NP1^21^, NP52, NP77, NP135, NP170, NP296, NP355^22^ and NP54^23^) were screened in pair-wise combinations for NP-dependent activation of an upstream activating sequence-regulated EGFP-2A-Gluc (UAS-EGFP-2A-Gluc, Fig. 1A) reporter (sandwich report system) in HEK293 cells (Fig. 1B). Previous research has demonstrated that these antibodies can inhibit the infection of Influenza A virus (IAV) or bind to NP of IAV, but the epitopes or affinity have not been determined. We screened for nanobodies pair could bind to NP simultaneously. A total of 64 AD/DBD-VHHs cross-pairs were evaluated and the results were presented in Fig. 1C.

**Figure 2:**
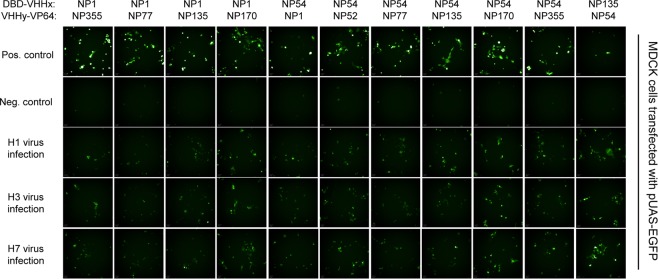
Sandwich report system applied to detect virus infection. MDCK cell expressed different nanobody pair was infected with different influenza virus 6 h after transfection. Cell co-expressed NP plasmid was positive control and cell co-transfected with vector was negative control. The fluorescence of cell was detected 48 h after infection.

Among all tests VHHs, the NP1 showed the best compatibility which yielded strong EGFP signals in either DBD- or AD-constructs when paired with anyone of the remaining 7 nanobodies. The DBD-NP54 construct also exhibited bright fluorescence when paired with most of AD-VHHs except itself. Although no fluorescence detected in most of DBD/AD-VHHs pairs when NP was absent, there were some weak EGFP fluorescence noted in some pairs such as DBD-NP135/AD-NP77, DBD-NP170/AD-NP1, DBD-NP135/AD-NP52 and DBD-NP296/AD-NP54 (Supplementary Fig. 1). These false-positive signals were possibly due to non-specific transcription activation derived from unexpected interaction between DBD and AD. Finally, we selected a total of 11 pairs for further evaluation in viral infection assay as shown in Fig. 2A. Madin-Darby Canine Kidney (MDCK) is the most widely utilized cell-line for IAV propagation. So we performed the infection assay in MDCK rather than HEK 293. Among these pairs, we observed MDCK cells which transfected with DBD-NP54/AD-NP170 presented better performance than others when the cells were infected by influenza viruses of subtype H1, H3 or H7 (Fig. 2). To test whether the fluorescence is specific to virus infection, we additionally performed a neutralization test on DBD-NP54/AD-NP170 reporter transfected MDCK cells during H3 virus infection. As the results shown in Supplementary Fig. 2,A,B, the fluorescence signal was significantly reduced when a neutralization antibody was used to block virus infection. This data suggested this new system could be used in anti-influenza A neutralization assay.

### Development and optimization of an all-in-one NP reporter plasmid

**Figure 3:**
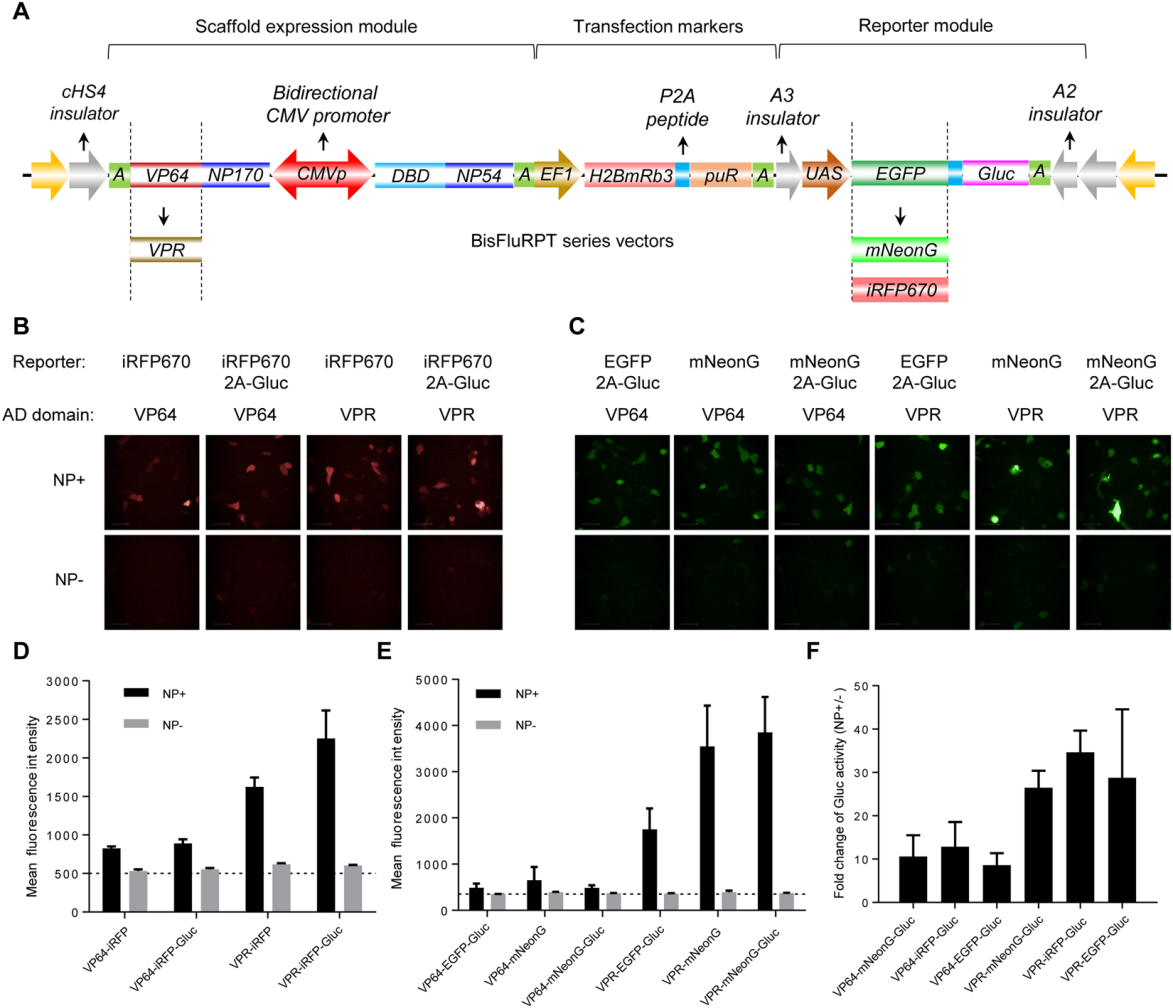
Optimization of the sandwich report system. (**A**) The schematic diagram of new constructed plasmids contained all elements need for sandwich report system. The replacement of report gene or transactivation was also showed. The new constructed plasmids expressed different fluorescent protein or activation domain were co-transfected with NP or vector. The iRFP fluorescence (**B,D**) and green fluorescence (**C,E**) was detected 48 h after transfection. The Gluc activity in supernatant was detected 48 h after transfection (**F**).

Although the sandwich report system described above is able to detect the intracellular NP proteins, the sensitivity of this system may be further improved therefore enabling detection of low-levels of influenza A infection. Several approaches were adopted to optimize the sensitivity of the sandwich report system based on the pair of DBD-NP54/AD-NP170. Firstly, the cassettes of DBD-NP54, AD-NP170 and UAS-EGFP-2A-Gluc were assembled into a single PiggyBac transposon plasmid (designated BisFluRPT-EGluc) to simplify the system. The fusion constructs of DBD-NP54 and AD-NP170 were driven by a constitutively active bidirectional CMV promoter. To minimize unexpected transactivation of reporter genes, the UAS-EGFP-2A-Gluc was bracketed with two potent enhancer-blocking insulators (A2 and A3^24^). In addition, an expression cassette of the EF1 promoter driving red fluorescent protein (mRuby3) and self-cleaving P2A peptide linked puromycin resistance gene (PuroR) was also introduced into the reporter plasmid (Fig. 3A) for indication and selection of successfully transfected cells. Secondly, we tested two new fluorescent proteins, mNeonGreen and iRFP670 for alternatives of EGFP. The mNeonGreen was engineered from yellow fluorescence protein (LanYFP) and is up to three times brighter than EGFP *in vitro*^25^. The iRFP670^26^,a near-infrared fluorescent protein, was also evaluated because of its plausibility to detect NP expression *in vivo*. Secretable Gaussia luciferase (Gluc) was selectively fused to the C-terminal of fluorescent proteins with a 2A peptide. Thirdly, we tried to replace the VP64 activation domain (tetrameric VP16) to a newly developed tripartite activator, VP64-p65-Rta (VPR)^27^ to increase the reporter expression. The systematical comparisons of fluorescence proteins and activation domains in a single plasmid system were shown in Fig. 3B–F. The fluorescence of reporters having iRFP is showed in Fig. 3B,D. The fluorescence of reporters having EGFP or mNeoGreen is showed in Fig. 3C,E. Figure 3F shows the fold change of luciferase activity (Gluc). As expected, the mNeonGreen and iRFP670 reporter showed improvement to some degree than EGFP-based reporter, but not very significantly. By contrast, the reporter signals, either fluorescence of fluorescent proteins or secreted Gluc, were dramatically increased for the plasmids which had the replacement of VP64 with VPR. As the VPR replacement didn’t result in a noise signal increasing in NP-absent cells, the new reporter with this modification showed a greatly improved (approximately 3–4 folds) signal-to-noise ratio (SNR).

### Preliminary evaluation of the optimized all-in-one reporter for living cell sensing of intracellular NP proteins

**Figure 4:**
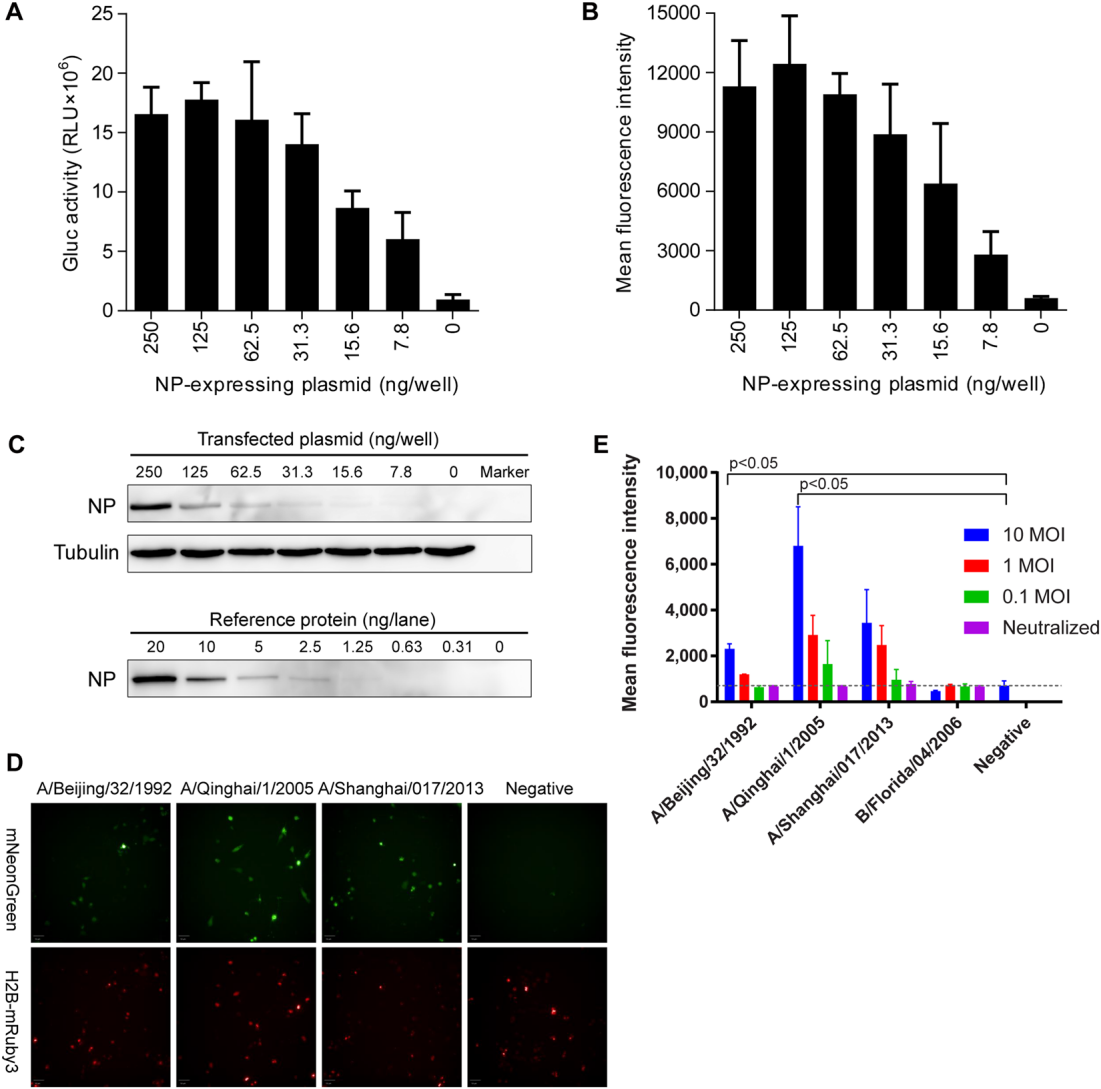
Application of the optimized sandwich report system for NP detection. The Gluc activity in supernatant (**A**), mean fluorescence intensity (**B**), and NP protein level (**C**) of MDCK cell expressed sandwich report gene (BisFluRPT-VPR-mNGluc) which co-transfected with different amount of NP-expressing plasmid. For upper panel of (C), the cell was lysed and NP expression level was detected by western blot and the level of tubulin was also detected as loading control. For the lower panel of (C), a series of dilutions of the recombinant NP protein were also detected by western blot as reference. The entire images of western blots were presented in Supplementary Fig. 3A–C. (**D**) Fluorescent images of MDCK cell expressed sandwich report gene (BisFluRPT-VPR-mNGluc) was infected with 3 subtypes of influenza A virus (MOI=10). (**E**) Quantitative analyses of mean fluorescence intensity of MDCK reporter cell infected with different influenza viruses, including A/Beijing/32/1992 (H3), A/Qinghai/1/2005 (H5), A/Shanghai/017/2013 (H7) and influenza B B/Florida/04/2006 at 3 different MOI (10, 1, 0.1). Infection neutralization assay was performed at the group of MOI=1 with neutralizing antibodies of FI6 (influenza A) or 12G6 (influenza B), in comparison to control antibody of 8G2.

We further tested the performance of the optimized all-in-one reporter (BisFluRPT-VPR-mNGluc) for living cell detection of intracellular NP proteins either derived from plasmid-transfections or various influenza A viruses infection. The sensitivity of the optimized sandwich report system was firstly tested by co-transfecting the BisFluRPT-VPR-mNGluc with different amounts (2-fold serial dilutions from 250 ng/well to 7.8 ng/well) of NP-expressing plasmid. As expected, our reporter plasmid generated mNeonGreen fluorescence (Fig. 4B) and secreted Gluc (Fig. 4A) in a dose-dependent manner with intracellular NP levels. Even when the NP-expressing plasmid was transfected with an extremely low dosage of 7.8 ng/well, of which the intracellular NP protein was undetectable by western blot (Fig. 4C), the reporter still resulted an over 20-fold signal increasing than NP-absent control cells. Moreover, we also performed infection and neutralization assays of various influenza A viruses to the reporter. The BisFluRPT-VPR-mNGluc transiently transfected MDCK cells were challenged with three subtypes of influenza A including H3 (A/Beijing/32/1992), H5 (A/Qinghai/1/2005) and H7 (A/Shanghai/017/2013) and a Flu B virus (B/Florida/04/2006, as a control) at multiplicities of infection of 10, 1 and 0.1 PFU. As the results shown in Fig. 4E, the control Flu B virus infection didn’t yielded any significant fluorescence increasing suggested a good specificity of this reporter. For the viruses of A/Beijing/32/1992 and A/Qinghai/1/2005, reporter transfected-cells showed significant increase in mNeonGreen fluorescence intensity (p < 0.05). Cell infected with virus of A/Shanghai/017/2013 also see slight increase in fluorescence. For all tested influenza A viruses (at MOI = 1), neutralization antibody of FI6 nearly completely blocked fluorescence (Fig. 4D,E), which suggested the reporter signals were really generated from intracellular NP proteins derived from virus infections. To test the response of this reporter system to different concentration of neutralizing antibodies, we used different concentration of FI6 antibody to neutralize the infection of virus A/Qinghai/1/2005 (H5). The fluorescence of cell (Supplementary Fig. 4B) and RNA level (Supplementary Fig. 4A) in cell lysate were both detected. The fluorescence of cell is positively correlated to RNA level (P < 0.0001, Supplementary Fig. 4C). This result makes it more convincing that this all in one reporter system could be applied to screen for IAV inhibitors.

## Discussion

Living cell viral infection reporter systems are indispensable tools for either basic researches regarding viral life cycle or antiviral drug developments. Such reporters usually allow scientist to visualize or quantify virus infection via fluorescence microscope-based imaging and/or luminometer, thereby enables convenient and high-throughput assays. For influenza virus, segmented viral genome provides opportunity to tag various segments with fluorescent or luminescent reporter genes. Several studies have played efforts to develop genetics-modified viruses carrying reporter genes of fluorescence and/or bioluminescent proteins. As early as 2004, Kittel *et al*. described the strategy to fuse GFP to a C-terminal truncated viral non-structural 1 (NS1) protein to generate recombinant GFP-reporting virus. However, recombinant influenza A virus only normally replicates in IFN-deficient Vero cells and protein kinase R (PKR) knockout mice^28^. Manicassamy *et al*. modified the NS gene to express NS1-GFP and nuclear export protein (NEP) in a single polyprotein via a self-cleaving 2A peptide, allowing functional NEP releasing from the translation of the upstream NS1-GFP fusion construct^29^. Such NS1-GFP virus generated GFP signals in its infected-cell both *in vitro* and *in vivo*, but showed attenuated infectivity when compared with wild-type virus. Avilov *et al*. utilized “split-GFP” to develop replication-competent influenza A reporter virus. In their system, the 11th beta-sheet of GFP protein (about 16aa) was fused to viral PB2 protein, fluorescence generation of GFP reconstitution occurs in cells-expressing GFP1-10 via protein trans-complementing^14^. More recently, Fukuyama *et al*. describe strategy to produce multi-spectral fluorescent reporter influenza viruses (designated color-flu) via incorporating of fluorescent proteins of different colors (EGFP, ECFP, mCherry and Venus) into NS1 gene of various virus strains^16^. For *in vivo* influenza infection, Pan *et al*. constructed bioluminescent influenza A virus by inserting Gaussia luciferase encoding sequence into the NA segment via 2A peptide ligation^30^. Moreover, Karlsson *et al*. utilized another bioluminescent reporter NanoLuc-based and PA gene-modified virus for real-time monitoring of the influenza virus infection, transmission and protection in ferrets^31,32^.

Overall, various recombinant fluorescent or luminescent reporter-expressing influenza A viruses have been generated by reverse genetics techniques for antiviral researches. However, indicating viral infection via this strategy is only applicable for limitative genetics engineered viruses but not for unmodified wild type viruses. As the influenza gene segments are small and reporter gene insertions usually affect the efficiencies of virus packaging and replication, most of recombinant reporter viruses were attenuated and required further adaptation in host cells or animals. Moreover, some studies had noted that the inserted reporter gene might be easily lost during continuous replication^33^. To address these issues, our study developed a universal reporter system for living cell sensing influenza A virus infection, which does not require any modification on virus. Enlightened by the study of Tang *et al*. regarding utilization of GFP as a scaffold for inducing formation of transcription reporter modular based GFP nanobodies^20^, we modified the system as a biosensor to detect intracellular influenza A NP protein for the first time. In our new reporter system, two NP-specific nanobodies, one fused to a DBD (DNA binding domain) and the other fused to a transactivation domain, were used for double-sandwich capture of NP. In UAS-reporter plasmid transfected cells, the presence of intracellular NP can induce the formation of sandwich immune complex and thereby formatted a hybrid transcription factor to initial the expression of UAS downstream reporter gene. We selected several nanobodies reported by previous studies for scaffold binder in this system. The result in Fig. 1C indicates that some nanobody pairs could induce high expression level of report gene while some nanobody pairs could hardly induce the expression of report gene. The epitopes of these nanobodies may determine this difference. Although epitopes of nanobodies used here hasn’t been determined, we can find that most of the self-paired DBD/AD combinations did not show satisfactory reactivity. It has been demonstrated that NP1 didn’t compete with NP52, NP77, NP135, NP170, NP296 or NP355 to bind NP and the combinations of NP1 and other VHHs all show high fluorescence in this result. From the theoretical and practical results, epitopes of nanobody pair would determine the appliance of this reporter system. The affinity of nanobodies may also influence the sensitivity of this report system, but it still remains to be confirmed.

And the results from transfections and infections (Figs 1C and 2) demonstrated that a pair of DBD-NP54/AD-NP170 could recognize different influenza A subtypes, at least including H1 H3 and H7. However, infection assay as shown in Fig. 4E showed different detection sensitivities of this pair for various influenza A infection suggested potential difference of various subtype on NP-expressing level during infection course. On the other hand, the difference also possibly attributed to the binding difference of NP54 or NP170 against NP of various subtypes. Although any two high-affinity binders (including nanobodies and single-chain variable fragments) which can bind NP protein simultaneously would be theoretically applicable for this system, some anti-NP nanobodies with potent blocking effects for nuclear import of vRNPs and influenza viral mRNA transcription (such as NP1) should be excluded to minimize the intrabody-mediated interference on viral infection dynamic.

To improve the convenience of this system, we develop an all-in-one plasmid which contained expression cassettes of DBD-NP54 and AD-NP170, UAS-reporter module and dual selection makers (mRuby3 and puromycin resistance gene) in a PiggyBac vector. This new vector may allow rapid generation of stable reporter cell lines for scalable applications via co-transfection with a PiggyBac transposase, and enrichment of cells carrying insertions through mRuby3-activated cell sorting and puromycin resistance selection. More importantly, we found a replacement of original VP64 transactivation domain with VPR appeared greatly improved performance for the new reporter system. The VPR is a newly developed hybrid transactivation protein which fused p65 and Rta in tandem to VP64. Previous studies had demonstrated the dCas9-VPR had more potent gene expression activation activity than its parental dCas9-VP64 construct^27^. In our system, VPR showed a 3–4 folds increasing both for absolute signal intensity and SNR value thus provided enhanced sensitivity for NP detection. Further improvements of this reporter system focusing on the optimizations of DBD/DNA-binding motif elements, as well as on the introduction of intracellular signal amplification system (such as SunTag^34^) may provide more sensitive performance to detect low-level influenza A virus infection. On the other hand, constructing of transgenic animal carrying the reporter may support *in vivo* visualization of viral infection of various influenza A subtypes. The effects of most drugs, including antibodies and small molecules, vary between *in vitro* and *in vivo*. Transgenic animal carrying the reporter having Gluc may provide a useful tool for screening and confirming the function of inhibitors *in vivo*.

Another advantage of our new reporter system was about its flexibility on developing biosensor for other intracellular targets. Theoretically, changing the NP54/NP170 to nanobodies (or other protein binders) against other targets may easily construct new specified reporter. Moreover, our preliminary results (not included in this paper) on intracellular viral DNA sensing via utilization of zinc finger arrays or dCas9/sgRNA suggested the potential of this system on living cell detection for non-protein biomolecules.

In summary, our study developed a novel reporter system for living cell sensing intracellular NP protein which allowed direct monitoring cell infections of unmodified various subtypes of influenza A viruses. The new reporter may provide a convenient and potent tool to facilitate anti-influenza drug discovery and vaccine development.

## Materials and Methods

### Cells

HEK293 (from ATCC, CRL-1573) and MDCK (kindly presented by prof. Honglin Chen from the University of Hong Kong) cells were grown in complete Dulbecco’s modified Eagle’s medium (DMEM) supplementaryed with 10% fetal bovine serum (FBS); penicillin, 100 units/mL; streptomycin 100 μg/mL and L-glutamine, 2 mM.

### Plasmids

The genes of 8 anti-NP nanobodies (NP1^21^, NP52, NP77, NP135, NP170, NP296, NP355^22^ and NP54^23^) were synthesized and ligated into plasmid pCAG-Gal4DBD-GBP2 (from Addgene, between NheI/NotI) and pCAG-GBP6-10gly-VPminx4 (from Addgene, between AgeI/NheI) by General Biosystems Company (Anhui, China). The sequence of these nanobodies are showed in Supplementary Table 1. The genes of EGFP and Gussia luciferase (Gluc) was ligated into pUAS-luc2 (Addgene #24343) between EcoRI/XbaI and 2 A peptide was used to link these two gene to get pUAS-EGFP-2A-Gluc. Similarly, the plasmids that contained all the components (mRuby3, DBD-NP54, AD-NP170 and UAS-EGFP-2A-Gluc or other report gene) was also synthesized by General Biosystems Company.

### Transfection

Lipofectamine® 3000 Transfection Reagent (L3000-015, Invitrogen) was used for cell transfection. The expression of proteins was detected 48hrs after transfection. Cell fluorescence images were collected by Opera Phenix High Content Screening System (PerkinElmer Inc, USA) and the fluorescence intensity was analyzed by the associated Harmony® imaging and analysis software. Intracellular Gaussia luciferase activities were detected by Pierce™ Gaussia Luciferase Flash Assay Kit (16159, Thermo Scientific).

### Western blot analysis

Whole-cell lysates of MDCK cells were electrophoresed through sodium dodecylsulfate polyacrylamide gels and transferred to Immobilon NC Transfer Membrane (HATF00010, Millipore). The membrane was then blocked with Blocking Buffer (Wantai, Beijing, China) and incubated with mouse anti-NP (19C10^18^, Innodx, Xiamen, China), or rabbit anti-tubulin (ab179513; Abcam) antibodies, followed by HRP-conjugated anti-mouse or anti-rabbit IgG antibody (Innodx, Xiamen, China), respectively. Chemiluminescence-based WB imaging were performed by using the SuperSignal West Femto Maximum Sensitivity Substrate (34095, Thermo Scientific). The specific protein bands were visualized by the ImageQuant LAS 4000 (GE Healthcare).

### Infection

Influenza virus strains (A/PR/8/1934, A/Beijing/32/1992, B/Florida/04/2006) were kindly provided by BEI Resources. The virulence attenuated virus strains of A/Qinghai/1/2005 and A/Shanghai/017/2013 were kindly presented by prof. Honglin Chen from the University of Hong Kong. The MDCK cells were seeded at a density of 20,000 cells per well in 96-well flat-bottom microplates. The influenza viruses of, A/Beijing/32/1992 (H3), A/Qinghai/1/2005 (H5), A/Shanghai/017/2013 (H7), or B/Florida/04/2006 (Flu B, served as a control virus) were serially diluted to match the multiplicity of infection (MOI) of 10, 1, and 0.1. Then, these influenza viruses were incubated with an excessive non-neutralizing antibody 8G2 (our lab) or neutralizing antibody (FI6^35^ for influenza A and 12G6^36^ for influenza B) at 37 °C for one hour. MDCK cells were incubated with virus or mixture of virus and antibody at 37 °C for one hour. The infected cells were then washed with PBS buffer for 3-times before refreshing culture medium. Infected cells were cultured at 37 °C with 5% CO2 for 48-hour before analyses. To test the response of this reporter system to different concentration of neutralizing antibodies, virus A/Qinghai/1/2005 (H5) was incubated with different concentration of FI6: high (50 μg/mL), middle (1 μg/mL), low (0.02 μg/mL) and control (no neutralizing antibody) at 37 °C for one hour and then added the mixture to MDCK cell. Influenza A virus nucleic acid assay kit (Shanghai ZJ Bio-Tech Co., RR-0051-01) was used to detect virus RNA.

## Supplementary information

Nanobody-based sandwich reporter system for living cell sensing influenza A virus infection

## Data Availability

All data in this study are available from the corresponding author upon reasonable request.

## Supplementary information

is available this paper at 10.1038/s41598-019-52258-7.
