# Supplementary material for: Nanobody-based sandwich reporter system for living cell sensing influenza A virus infection
Source: Sci Rep. 2019 Nov 4;9:15899. doi: 10.1038/s41598-019-52258-7 (PMC6828950; doi:10.1038/s41598-019-52258-7)
Supplement: Supplementary file 1 — Nanobody-based sandwich reporter system for living cell sensing influenza A virus infection [file 41598_2019_52258_MOESM1_ESM.docx]

**Nanobody-based sandwich reporter system for living cell sensing influenza A virus infection**

Jia-Li Cao^1,†^, Nicole Zhong^3,†^, Guo-Song Wang^1,†^, Ming-Feng Wang^1^, Bao-Hui Zhang^2^, Bao-Rong Fu^2^, Ying-Bin Wang^2^, Tian-Ying Zhang^1^, Ya-Li Zhang^1^, Kunyu Yang^4^, Yi-Xin Chen^1,*^, Quan Yuan^2,*^, Ning-Shao Xia^1, 2^

^1^ State Key Laboratory of Molecular Vaccinology and Molecular Diagnostics, National Institute of Diagnostics and Vaccine Development in Infectious Diseases, School of Life Sciences, Xiamen University, Xiamen, 361102, P.R. China.

^2^ State Key Laboratory of Molecular Vaccinology and Molecular Diagnostics, National Institute of Diagnostics and Vaccine Development in Infectious Diseases, School of Public Health, Xiamen University, Xiamen 361102, China.

^3^ Concordia International School Shanghai, 345 Huangyang Road Pudong, Shanghai China 201206, P.R. China.

^4^ Xiamen International Travel Healthcare Center, Xiamen, China.

^†^ These authors contributed equally to this work.

^*^ **Correspondence:**

Address requests for reprints to: Quan Yuan (yuanquan@xmu.edu.cn) or Yixin Chen ([yxchen2008@xmu.edu.cn](mailto:yxchen2008@xmu.edu.cn)), State Key Laboratory of Molecular Vaccinology and Molecular Diagnostics, Xiamen University, Xiamen 361102, People’s Republic of China. Fax: (86)-05922181258.


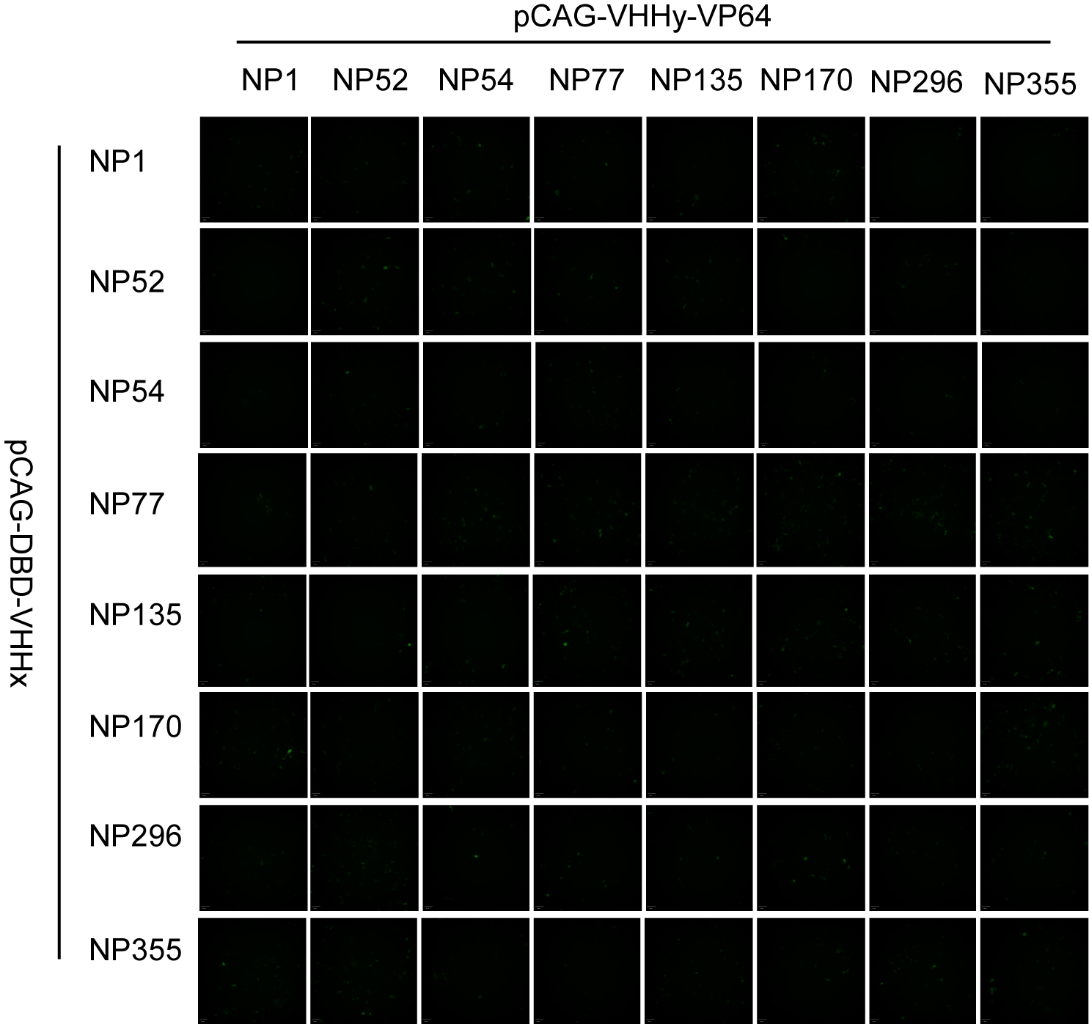


**Supplementary Figure 1**. Screening for nanobody pair suitable for NP detection. Different nanobody pair and report gene were co-transfected with vector as control in HEK293 cell. The fluorescence was detected 48h after transfection.


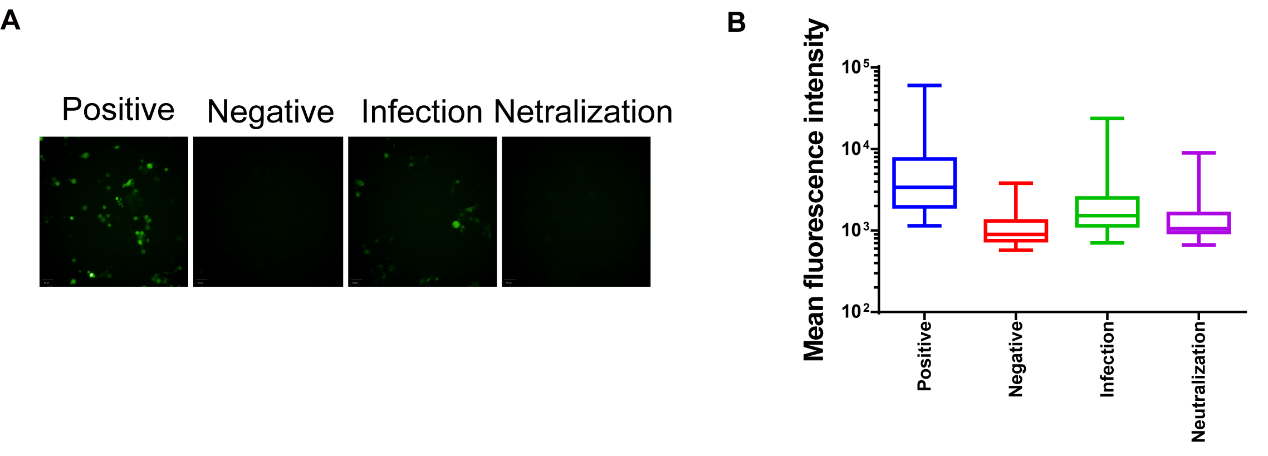


**Supplementary Figure 2.** Neutralization of influenza A virus infection. DBD-NP54 and VP64-NP170 was chose for neutralization experiment. H3 subtype of influenza A was used to infect transfected cell and antibody FI6 was used to neutralized the infection. The fluorescence was detected 48h after infection (A). The intensity of green positive cell was calculated and showed in (B).


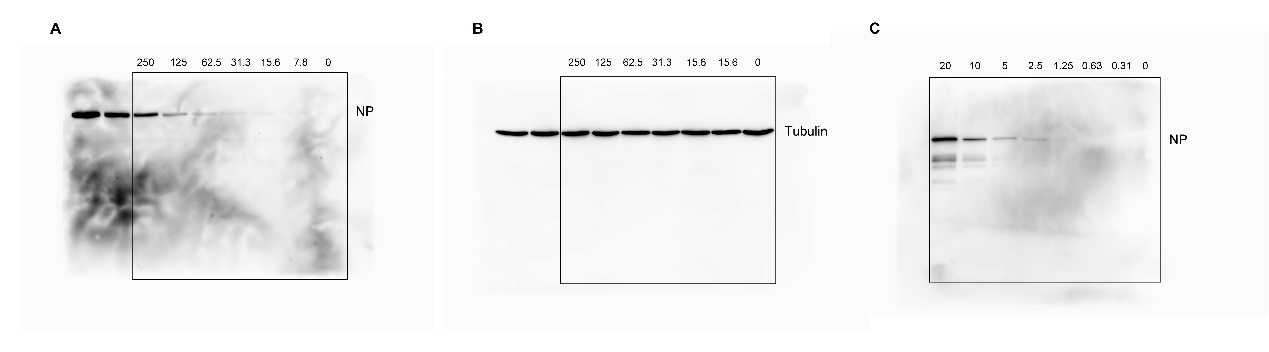


**Supplementary Figure 3.** The NP protein expression level of cell transfected different dose of NP plasmids. NP expression level was detected by western blot (A), the expression of tubulin was also detected as reference (B). C NP protein reference.


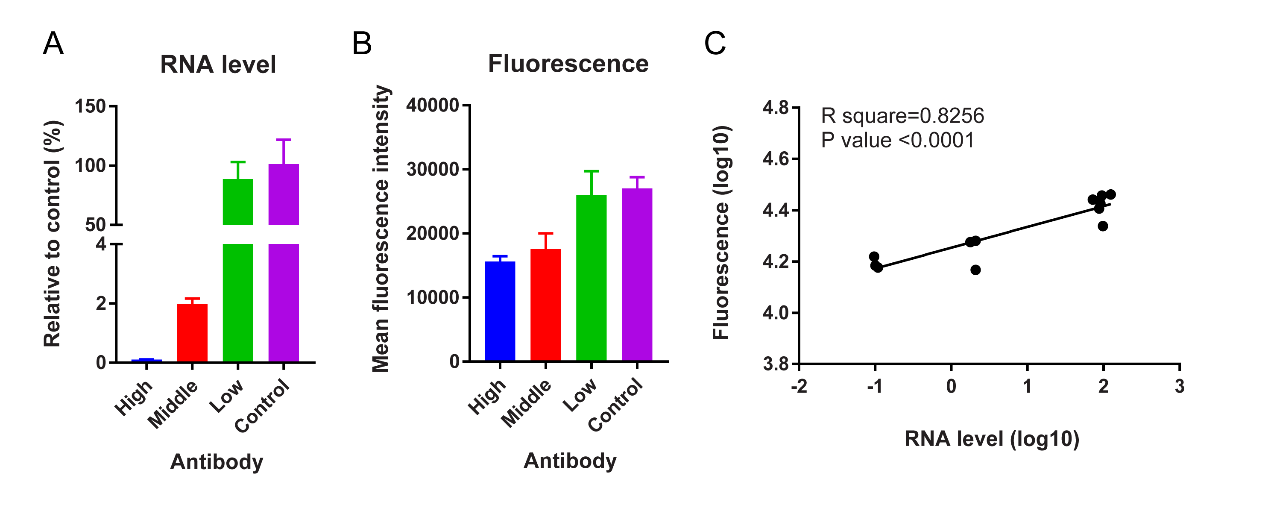


**Supplementary Figure 4.** The RNA level in cell lysate (A) and fluorescence (B) of cell infected with A/Qinghai/1/2005 (H5) and neutralized with different concentration of antibody (compare to control group). C The correlation between fluorescence (log10) and RNA level (log10).

**Supplementary table1.** The sequence of nanobodies used in this research

| name | sequence |
| --- | --- |
| Np1 | ATGGCCCAGGTGCAGCTGCAGGAGAGCGGCGGCGGCCTGGTGCAGGCCGGCGGCAGCCTGCGCCTGACCTGCGCCCTGAGCGAGCGCACCAGCACCAGCTACGCCCAGGGCTGGTTCCGCCAGCCCCCCGGCAAGGAGCGCGAGTTCGTGGCCAGCCTGCGCACCCACGACGGCAACACCCACTACACCGACAGCGTGAAGGGCCGCTTCACCATCAGCCGCGACAACGCCGAGAACACCCTGTACCTGCAGATGAACAGCCTGAAGACCGAGGACACCGCCGTGTACTACTGCGCCGCCAGCCTGGGCTACAGCGGCGCCTACGCCAGCGGCTACGACTACTGGGGCCAGGGCACCCAGGTGACCGTGAGCAGCTAA |
| Np52 | ATGGCCCAGCTGCAGCTGGTGGAGAGCGGCGGCGGCCTGGTGCAGGCCGGCGGCAGCCTGCGCCTGAGCTGCGCCGCCAGCGGCCTGAGCCTGAGCGACTACGCCATGGGCTGGTTCCGCCAGGCCCCCGGCAAGGAGCGCGAGTTCGTGAGCGGCATCGGCTGGAACGGCCTGCGCATCGCCTACGCCGACTTCGTGAAGGGCCGCTTCACCATCAGCCGCGACAACGCCAAGAACACCGCCTACCTGCAGATGAACAGCCTGAAGCCCGAGGACAGCGCCGTGTACTACTGCGCCGCCGCCCGCAGCGAGTGGGGCAGCCGCGCCGTGTACGACTACTGGGGCCAGGGCACCCAGGTGACCGTGAGCAGCTAA |
| Np54 | ATGGCCCAGGTGCAGCTGCAGGAGAGCGGCGGCGGCCTGGTGCAGGCCGGCGGCAGCCTGCGCCTGAGCTGCGCCGCCAGCGGTCGCACCCTGAGCAGCTACGCCATGGGCTGGTTCCGCCAGGCCCCCGGCAAGGAGCGCGAGCTGGTGAGCGCCATCAGCTGGAGCGGCCTGAGCACCTACTACGAGGACAGCGTGAAGGGCCGCTTCACCATCAGCCGCGACAACGCCAAGAACACCATGTACCTGCAGATGAACAGCCTGAAGCCCGAGGACACCGCCATCTACTACTGCGCCGCCGACATCGGCTGGCCCCTGCGCGCCGACAGCGGCAGCTGGGGCCAGGGCACCCAGGTGACCGTGAGCAGCTAA |
| Np77 | ATGGCCCAGGTGCAGCTGGTGGAGACCGGCGGCGGCCTGGTGCAGCCCGGCGGCAGCCTGCGCCTGAGCTGCGCCGCCAGCGGCTTCACCTTCAGCGACTACTACATGACCTGGGTGCGCCAGGCCCCCGGCAAGGGCCCCGAGTGGGTGAGCTGGATCAACAGCCGCGGCACCGGCACCGGCTACGCCGACAGCGTGCAGGGCCGCTTCACCATCAGCCGCGACAACGCCAAGAACACCCTGTACCTGCAGATGGACAGCCTGCGCCCCGAGGACACCGGCCTGTACTACTGCGCCCGCGGCATGATCCACATCGAGACCACCCTGCCCCAGGCCCGCGGCCAGGGCACCCAGGTGACCGTGAGCAGCTAA |
| Np135 | ATGGCCCAGCTGCAGCTGGTGGAGAGCGGCGGCGGCCTGGTGCAGCGCGGCGGCAGCCTGCGCCTGAGCTGCGCCGCCAGCGGCGGCACCGTGAGCACCATCGACATGGGCTGGTTCCGCCAGGTGCCCGGCAAGGAGCGCGAGTTCGTGGCCGGCATGAGCAGCAGCGGCCACGTGACCAGCACCGGCGACAGCGTGAAGGGCCGCTTCACCATCAGCAAGGACAACGCCAAGAACACCGTGTACCTGCAGATGAACGACCTGAAGCCCGAGGACACCGCCGTGTACTACTGCGCCAGCGGCAACTGGAACAGCCGCGCCCGCGAGTACGACAGCTGGGGCCAGGGCACCCAGGTGACCGTGAGCAGCTAA |
| Np170 | ATGGCCCAGCTGCAGCTGGTGGAGAGCGGCGGCGGCCTGGTGCAGGCCGGCGGCAGCCTGCGCCTGAGCTGCGCCGCCAGCGGCTTCACCTTCCGCAACAGCGCCATGAGCTGGGTGCGCCAGGCCCCCGGCAAGGGCCTGGAGTGGGTGAGCACCATCAACACCGGCGGCAGCGGCGCCGCCTACGCCGACGCCGTGGCCGGCCGCTTCACCATCAGCCGCGACAACGCCAAGAACACCCTGTACCTGCAGATGAACAGCCTGAAGCCCGAGGACACCGCCGTGTACTACTGCAAGGACAGCGACTTCGGCCAGCGCATCTTCACCACCCGCGGTCGCGACTGGGGCCAGGGCACCCAGGTGACCGTGAGCAGCTAA |
| Np296 | ATGGCCCAGGTGCAGCTGGTGGAGAGCGGCGGCGGCGTGGTGCAGGCCGGCGGCAGCCTGCGCCTGAGCTGCGCCGCCAGCGGCTTCACCTTCCGCGACTACGCCATGCACTGGGTGCGCCAGGCCCCCGGCAAGGGCCTGGAGTGGGTGAGCGCCACCAACGCCGGCGGCGCCCTGACCGCCTACAGCGACAGCGTGGCCGGCCGCTTCACCATCAGCCGCGACAACGCCAAGAACACCCTGTACCTGCAGATGAACAGCCTGAAGCCCGAGGACACCGCCGTGTACTACTGCAGCAAGAACGACTTCGGCCAGCGCCTGTTCACCAGCCGCGGTCGCGACTGGGGCCAGGGCACCCAGGTGACCGTGAGCAGCTAA |
| Np355 | ATGGCCCAGCTGCAGCTGGTGGAGACCGGCGGCAACCTGGTGCAGGCCGGCGGCAGCCTGCGCCTGAGCTGCGCCGCCAGCGGCTTCACCTTCCCCAACTTCGACATGAGCTGGGTGCGCCAGGCCCCCGGCAAGGGCCTGGAGTGGGTGAGCAGCATCAACACCCGCGGCAAGATCGAGGCCTACGCCGACGCCGTGAAGGGCCGCTTCACCATCAGCCGCGACAACGCCGCCAACACCCTGTACCTGCGCATGGACAGCCTGAAGCCCGAGGACACCGCCGTGTACTTCTGCGTGAAGAGCGACTTCGGCCAGCGCATCTTCACCACCCGCGGTCGCGACTGGGGCCAGGGCACCCAGGTGACCGTGAGCAGCTAA |
